# A Narrative Review of New Emerging Urological Interventions for Benign Prostatic Hyperplasia: HoLEP, Rezum, and Aquablation

**DOI:** 10.7759/cureus.97502

**Published:** 2025-11-22

**Authors:** Vyshnavi Sathish

**Affiliations:** 1 Urology Department, Torbay and South Devon NHS Trust, Torquay, GBR

**Keywords:** aquablation, bph, holep, rezum, turp

## Abstract

Benign prostatic hyperplasia (BPH) is more prevalent with increasing age, leading to presentations with lower urinary tract symptoms (LUTS). Transurethral resection of the prostate (TURP) remains the gold standard surgical treatment, but its limitations and complications have prompted the development of minimally invasive surgeries. This review evaluates the efficacy and safety of holmium laser enucleation of the prostate (HoLEP), water vapor therapy (Rezum), and robotic water-jet resection (Aquablation) in benign prostate surgery. A narrative review was conducted using PubMed and Google Scholar to identify current literature published between January 2000 and August 2025. Meta-analyses and randomized controlled trials (RCTs) were preferred, but all levels of evidence were considered for this article. Search terms included "benign prostatic hyperplasia", "BPH", "Rezum", "HoLEP", and "Aquablation". These emerging minimally invasive prostate surgeries show promising outcomes and have a role to play, depending on patient choices, surgical expertise, and anatomical factors. HoLEP provides good long-term outcomes and is non-inferior to TURP outcomes in International Prostate Symptom Score (IPSS) and maximum urinary flow rate (Qmax), particularly in larger prostates. Rezum can be performed in an outpatient setting and is effective in preserving sexual function. Aquablation is also effective in preserving sexual function and is suitable for larger prostates. In conclusion, surgical options for managing BPH are evolving technologies that are increasingly replacing the gold standard TURP. More multicenter RCTs comparing all techniques are required to ensure that the data are representative across all populations.

## Introduction and background

Benign prostatic hyperplasia (BPH) is a common urological condition in men that causes lower urinary tract symptoms (LUTS). It significantly impacts quality of life, leading to presentations in both emergency settings and outpatient urology clinics, contributing to a substantial healthcare burden. Histologically, BPH at the time of autopsy was 60% in men in their 60s, increasing to 80-90% in men older than 70 years of age. Pathologically, BPH is characterized by proliferation of stromal and epithelial cells in the transition zone of the prostate, surrounding the urethra, which leads to urethral compression and chronic LUTS. These symptoms are predominantly voiding symptoms, including hesitancy, poor flow, intermittency, and incomplete bladder emptying, though some storage symptoms of frequency and nocturia may be present as well. This is often due to bladder outlet obstruction, secondary to an enlarged prostate [1].

The first-line management of BPH is medical treatment with alpha blockers and 5-alpha-reductase inhibitors. However, surgical intervention may be required as symptoms progress or complications develop, such as refractory or high-pressure chronic retention, recurrent urinary tract infections, or bladder stones. Traditionally, transurethral resection of the prostate (TURP) has been the gold standard surgical intervention for BPH [1]. However, complications of TURP may include bleeding, TUR syndrome, urinary incontinence, urethral stricture, and retrograde ejaculation. It also has a high complication rate, with a re-treatment rate of 3.7% that increases by 1%-2% each subsequent year [2]. These limitations and advancements in technology have shifted the surgical intervention of prostatic surgery towards minimally invasive treatment options using laser and steam therapy [3]. Among these, holmium laser enucleation of the prostate (HoLEP), water vapor thermal therapy (Rezum), and robotic water jet resection (Aquablation) are increasingly used across the urological departments in the United Kingdom.

According to National Institute for Health and Care Excellence (NICE) guidelines, HoLEP is recommended particularly in specialist centers, alongside TURP, as an effective surgical option [4]. Rezum is also endorsed by NICE for its cost-effectiveness and suitability as a day-case procedure [5]. Aquablation is a newer robotic water-jet-based technique that avoids thermal energy and has gained attention for its promising safety profile and precision [6]. Given NICE approval and growing clinical adoption, this narrative review aims to evaluate the current evidence on the efficacy, safety, and clinical effectiveness of HoLEP, Rezum, and Aquablation in the management of BPH.

## Review

Methods

A narrative review was conducted to evaluate the current evidence on surgical interventions for BPH, focusing on HoLEP, Rezum, and Aquablation. Google Scholar and PubMed were used to retrieve papers published between January 2000 and August 2025. Search terms included "benign prostatic hyperplasia", "BPH", "HoLEP", "holmium laser enucleation", "Rezum", and "Aquablation". Systematic reviews and meta-analyses were preferred; however, all types of literature were used to allow for a broader interpretation of clinical practice. Only peer-reviewed, English-language papers involving patients with BPH were included.

HoLEP

The holmium:yttrium aluminium garnet laser (Holmium), with a wavelength of 2,140 nm, was one of the first lasers that was successfully used for BPH. It aims to remove the entire adenoma from the surgical capsule and displace it into the bladder. A transurethral soft tissue morcellator is then used for removal. The HoLEP technique ensures the removal of prostatic tissue from the transition zone, reducing the need for repeat surgery. By removing the entirety of the prostatic tissue in the transition zone, it is equivalent to an open prostatectomy [7].

Systematic reviews and meta-analyses of the efficacy and safety of HoLEP highlighted that HoLEP is, in fact, non-inferior to other interventions in terms of operative time, enucleation, morcellation, or catheterization. Compared to TURP, it has better operative hemostasis, as evidenced by lower perioperative hemoglobin decrease, resulting in shorter recovery, hospital stay, and catheterization time. There was also a significant improvement in peak flow rate (Qmax) and patient-reported quality of life [8].

Qian et al. conducted a systematic review comparing HoLEP with bipolar TURP and found comparable functional outcomes for Qmax, post-voidal residual (PVR), and International Prostate Symptom Score (IPSS). This paper highlighted that HoLEP was non-inferior to TURP and that both procedures were safe [9]. Furthermore, Elmansy et al. conducted a long-term follow-up (10 years) of 949 patients who underwent HoLEP. They examined PVR, Qmax, and the mean IPSS at one month, one year, and 10 years following the intervention. The study found that HoLEP patients did not have worsening PVR and Qmax even at 10-year follow-up. In fact, the IPSS score improved with time at one year and 10 years. Re-operation rates due to residual adenoma were minimal (0.8-1.5%) at one month, one year, and 10 years, demonstrating that HoLEP was an effective treatment option with long-term durability [10].

Rezum

Rezum is another minimally invasive transurethral water vapor therapy for BPH. This technique uses thermal energy for treatment. It is preferred for patients who are suitable for an outpatient setting, with the added benefit of an improved chance of preserving sexual function. Rezum uses convective heat transfer that utilizes the thermodynamic properties of water. The Rezum system uses a radiofrequency (RF) generator and a single-use transurethral standard cystoscope lens. RF current is applied to an inductive coil heater, which produces water vapor, a form of thermal energy. This water vapor is delivered to the transition zone of the prostate through a retractable vapor needle via emitter holes in the cystoscope in nine-second bursts. Upon contact with body tissue, water vapor condenses into a liquid, causing targeted tissue necrosis. Magnetic resonance imaging and histological testing have been used post-intervention to assess the efficacy of Rezum, demonstrating that it is clinically safe, as tissue necrosis is targeted while preserving non-treated tissue around the area. Multiple studies have been conducted since the Food and Drug Administration (FDA) approval of Rezum [11].

McVary et al. conducted a multicenter randomized controlled trial (RCT) to assess the five-year outcomes of Rezum in men with BPH. The study demonstrated durable symptom relief, with mean IPSS scores reducing from 22 to 10 with quality of life improving by 45%. There were no reports of device- or procedure-related adverse effects on sexual function, and the re-treatment rate was 4.4%. Although long-term data remain limited, Siqueira et al. recently published a multicenter study assessing the long-term outcomes of Rezum. The study looked at 712 patients with a baseline prostate volume of 74.1 cc. PVR and IPSS score decreased significantly with significant Qmax improvement from baseline to 36 months [12,13]. These findings reinforce Rezum as a safe, effective, and durable minimally invasive option for patients seeking symptom improvement while preserving sexual function.

Aquablation

Aquablation is an emerging prostate surgery for BPH. It is surgeon-tailored, with 3D ultrasound guidance and robotic-assisted resection of the prostate using a high-velocity water jet. Aquablation requires three main components: a conformal planning unit, a robotic 24F handpiece, and a console. It can be done under spinal or general anesthesia. Following resection, selective cautery is used to achieve hemostasis [14]. The WATER trial was a pivotal study in the use of aquablation, comparing the safety and efficacy of aquablation and TURP for the treatment of BPH. The trial was a multicenter, double-blinded RCT that primarily assessed the IPSS score at six months and the development of grade 1 or higher complications according to the Clavien-Dindo classification.

The WATER trial showed that, although the total operative time for aquablation and TURP was similar, resection time was significantly shorter with aquablation compared to TURP (4 vs. 27 minutes). Aquablation was non-inferior to TURP, and the rate of anejaculation was significantly lower in the aquablation group. Aquablation was superior to TURP in larger prostates [15]. The WATER II trial was conducted to assess safety and efficacy in patients with large-volume prostates (80-150 mL). This multicenter international trial showed that only 6% of patients required perioperative transfusions, and the mean length of stay after the procedure was 1.6 days. It concluded that aquablation was safe in men with large prostates [16]. Both trials demonstrated a significant improvement in IPSS and Qmax from baseline to 60 months. Moreover, more than 90% of patients in both trials were both BPH medication-free and free from requiring surgical intervention at five-year follow-up [17]. The mechanisms, outcomes, and advantages of HoLEP, Rezum, and Aquablation in the surgical management of BPH are summarized in Table 1 below.

**Table 1:** Comparative Summary of HoLEP, Rezum, and Aquablation in the Surgical Management of Benign Prostatic Hyperplasia (BPH) Data adapted from [4-19].

	Benign Prostate Surgery		
	HoLEP	Rezum	Aquablation
Mechanism of action	Uses a holmium laser of 60–80 watts, performed through a resectoscope [4,18].	Uses water vapor steam therapy, destroys excess prostate tissue, injected through a single-use device attached to a urological endoscope [5].	Robotic therapy, resection performed using a water-jet, performed through a robotic handpiece placed within the urethra (transurethral water-jet ablation), uses system software to help map the prostate [19].
Ideal prostate size	Larger prostates up to 100 grams [18].	30–80 grams [5].	Potential to be used in all sizes of prostate and can be used in large and complex prostates [19].
Advantages	Shorter operative time compared to TURP, reduced bleeding and reduction in transfusion rates, fewer TUR syndromes, shorter post-operative stay, and reduced catheterization time [18].	Significant cost-effectiveness (approximately £737) compared to TURP, avoids damage to nerves, erectile dysfunction is rare, same-day discharge, can be done under local anesthesia, takes only 20 minutes [5].	Preserves erection and ejaculation [15,16,19].
Training requirements/disadvantages	Steep learning curve with significant endoscopic skill [4,18].	Easy to learn with minimal training requirements, availability of the procedure in local hospitals, and unsafe to be done as a day case if there is an increased bleeding risk, such as anticoagulation use [5,12,13].	Specific training required; however, there is a short learning curve and a higher bleeding risk but this has been reduced with bladder neck cautery, very small risk of rectal perforation [15,16,19].
Clinical outcome improvements	Improvement in Qmax by 7.18 mL/s (comparable to TURP), mean PVR at one month and one year (45 mL; 57 mL), mean IPSS score at one month and one year (7.3; 3.8), re-operation rates at one year of 0.7% [8,10].	Improvement in Qmax from a baseline mean of 8.6 mL/s to 12.1 mL/s, PVR reduction from 134.9 mL to 38.5 mL at 36 months, IPSS score reduction from 22 to 9.8, re-treatment rate of 4.4% over a 4-year follow-up [12,13].	Reduced resection time comparative to TURP, mean length of stay—1.6 days, significant improvement in IPSS from baseline 23 to 7, Qmax improvement from baseline 9 mL/s to 17 mL/s at 60 months, minimal re-treatment rates [15,16].
Key studies	Sun et al. [8]; Elmansy et al. [10]; NICE Guidelines [4,18].	McVary et al. [12]; Siqueira et al. [13]; NICE guidelines [5].	WATER [15]; WATER II [16]; NICE Guidelines [19].

Discussion

It is important to acknowledge that this narrative review has limitations. While there are meta-analyses and some RCTs, there remains limited research directly comparing all three interventions (HoLEP, Rezum, and Aquablation) through large, multicenter RCTs that assess short-, mid-, and long-term outcomes. Additionally, this review may be limited due to selection bias and heterogeneity across studies.

BPH management is a growing field with emerging technologies. All three (HoLEP, Rezum, and Aquablation) seem to be safe, effective, and non-inferior to standard TURP. Each intervention should be guided by individual clinical factors such as prostate volume, anatomical features, the need for an outpatient day-case procedure, and individual patient factors, including quality of life and desire to preserve ejaculatory function.

HoLEP is more suitable for patients with larger prostate volumes and has been proven to be safe and non-inferior to TURP and has demonstrated significant symptom improvement [9,18]. However, it is associated with higher rates of dysuria and transient incontinence. However, evidence suggests that transient incontinence rates are improving as surgical expertise and experience increase [20]. It also has a risk of retrograde ejaculation that may make it less suitable for younger patients [21]. Rezum can be offered in an outpatient setting, with preservation of sexual function with minimal anesthetic risks [5,22]. However, its durability is limited, with re-treatment rates seven times higher than those after TURP [23]. Aquablation offers a promising alternative for me with moderate to large prostates. It has a shorter learning curve, but histological evaluation of ablated tissue is limited [6,19]. Ejaculatory function is much better preserved with Aquablation. However, it has higher rates of hematuria and a small risk of rectal perforation [15,16,19].

All surgical interventions are emerging techniques requiring both surgeon expertise and institutional resources. This emerging data may result in more safe and effective choices being available to patients undergoing prostate surgery; however, this might be constrained by the cost and availability of trained surgeons [21]. According to NICE guidelines [6], the cost of aquablation, which requires advanced technology, is currently £2872 per patient. There are limited papers conducted in the UK directly comparing the cost-effectiveness of all three technologies, including TURP. However, NICE guidelines have acknowledged that HoLEP may be used by only 25% of urologists, as the cost of holmium laser and equipment may limit its use across all NHS trusts [24]. In contrast, Rezum is cost-effective by £737 compared to TURP [5].

Ultimately, with growing evidence and new technologies, there needs to be an open discussion of all available treatment options for BPH to enable shared, patient-centric decision-making.

## Conclusions

In conclusion, while TURP remains the gold standard intervention for the surgical management of BPH, new technologies have been adapted that are proven to be effective and safe. HoLEP, Rezum, and Aquablation have all been approved by NICE and have shown significant improvements in Qmax and IPSS scores, with a good safety profile.

Each of these surgical interventions has its own advantages and limitations; therefore, the choice of procedure should be individualized with consideration of patient preference and surgical availability. This narrative review has summarized key study findings; however, further multicenter RCTs are required to compare all surgical prostate interventions. With new evidence, these procedures may redefine the standard of care in bladder outlet flow obstruction surgery.
